# Immune Modulation with Nanodiscs: Surface Charge Dictates Cellular Interactions and Activation of Macrophages and Dendritic-like Cells

**DOI:** 10.3390/ijms26115154

**Published:** 2025-05-28

**Authors:** Scarlett Zeiringer, Martina Derler, Marion Mussbacher, Tatjana Kolesnik, Eleonore Fröhlich, Gerd Leitinger, Dagmar Kolb, Sarah Tutz, Carolyn Vargas, Sandro Keller, Eva Roblegg

**Affiliations:** 1 Institute of Pharmaceutical Sciences, Pharmaceutical Technology and Biopharmacy, University of Graz, Universitätsplatz 1, 8010 Graz, Austria; scarlett.zeiringer@uni-graz.at; 2 Institute of Pharmaceutical Sciences, Pharmacology and Toxicology, University of Graz, Humboldtstraße 46/II, 8010 Graz, Austria; martina.derler@uni-graz.at (M.D.); marion.mussbacher@uni-graz.at (M.M.); 3 Center for Medical Research, Medical University of Graz, Stiftingtalstraße 24/1, 8010 Graz, Austria; tatjana.kolesnik@medunigraz.at (T.K.); eleonore.froehlich@medunigraz.at (E.F.); 4 BioTechMed-Graz, 8010 Graz, Austria; gerd.leitinger@medunigraz.at (G.L.); sandro.keller@uni-graz.at (S.K.); 5 Gottfried Schatz Research Center, Division of Cell Biology, Histology and Embryology, Research Unit Electron Microscopic Techniques, Medical University of Graz, Neue Stiftingtalstraße 6/V, 8010 Graz, Austria; 6 Core Facility Ultrastructure Analysis, Neue Stiftingtalstraße 6/II, 8010 Graz, Austria; dagmar.kolb@medunigraz.at; 7 Gottfried Schatz Research Center for Cell Signaling, Metabolism and Aging, Division of Cell Biology, Histology and Embryology, Medical University of Graz, Neue Stiftingtalstraße 6/II, 8010 Graz, Austria; 8 Biophysics, Institute of Molecular Biosciences (IMB), NAWI Graz, University of Graz, Humboldtstr. 46/III, 8010 Graz, Austria; sarah.tutz@uni-graz.at (S.T.); carolyn.vargas@uni-graz.at (C.V.); 9 Field of Excellence BioHealth, University of Graz, 8010 Graz, Austria

**Keywords:** monocyte differentiation, macrophages, immature dendritic-like cells, in vitro immune model, nanodiscs, immune response modulation, immune cell activation

## Abstract

The immunological barrier is among the most significant barriers in vivo. Macrophages and dendritic cells play a crucial role in immune responses, involving phagocytosis, antigen presentation, and triggering adaptive responses. Nanoscale drug-delivery vehicles, such as polymer-encapsulated lipid-bilayer nanodiscs, are of particular interest in the development of new therapeutic approaches, but require well-characterized human in vitro cell models. To this end, the present study differentiated human monocytes into two distinct states, resting macrophages and immature dendritic-like cells (iDCs). These cells served as model systems to assess the efficacy of lipid-bilayer nanodiscs encapsulated by anionic glyco-DIBMA (diisobutylene–maleic acid) or electroneutral sulfo-DIBMA polymers. Nanodisc–cell interaction studies—including cell viability, reactive oxygen species production, cytokine release, particle uptake, and activation marker expression—demonstrated that immune responses depend sensitively on the cell type and polymer and thus on the surface charge of the nanodiscs. Sulfo-DIBMA nanodiscs induced minimal immune cell activation, accompanied by cytokine release and reduced uptake of the nanodiscs by immune cells. In contrast, glyco-DIBMA nanodiscs exhibited increased interactions with cells, elicited pro-inflammatory immune responses, and promoted iDC maturation. This involved co-stimulatory and antigen-presenting molecules, potentially leading to T-cell activation. These findings underscore the potential of glyco-DIBMA nanodiscs to modulate immune responses through receptor-specific interactions, paving the way for immunotherapeutic strategies.

## 1. Introduction

Biological barriers play a key role in maintaining homeostasis between an organism and its environment. They act as an interface to control the exchange of substances while protecting against pathogens and harmful agents. The main components of biological barriers are either endothelial or epithelial cell layers whose function is regulated by their microenvironment [1]. In addition, immune cells play an essential role in the defense against and response to exogenous factors, with monocytes, macrophages, and dendritic cells (DCs) forming the innate immune system and T and B cells constituting the adaptive immune system [2]. Innate immunity acts as the first line of defense by recognizing pathogens via pattern recognition receptors (PRRs) and initiating responses such as phagocytosis, cytokine release, and recruitment of immune cells (e.g., neutrophils). Antigen-presenting cells (APCs), especially DCs, link innate and adaptive immunity by presenting processed antigens on major histocompatibility (MHC) molecules to activate CD8+ T cells or CD4+ T helper (Th) cells, thereby orchestrating downstream immune responses [3,4].

Macrophages and DCs are often key targets in drug development due to their abundance and immunomodulatory role. In in vitro studies, they are predominantly differentiated from monocytic cell lines, with murine (e.g., RAW 264.7) and human (e.g., THP-1) cells being used [5]. Due to its physiologically relevant origin, the latter is a well-established cell line that after differentiation exhibits typical characteristics of macrophage subtypes (i.e., M0, M1, M2) or immature and mature DCs [5,6,7,8]. This allows investigations in a variety of contexts, such as tumor research, inflammation, and infection, or research on the development of new drug candidates and nanosized drug-delivery systems (NDDSs). Thereby, NDDSs come into contact with immune cells regardless of their route of application. This contact is crucial for the fate of NDDSs, influencing biodistribution, potential elimination, and stimulation or suppression of an immune response [9,10]. The specific design of NDDSs allows for the modulation of immune responses, thus enabling their exploitation for targeted applications. An example of an NDDS is nanodiscs, which are disc-shaped, lipid-containing nanoparticles with typical sizes of 10–20 nm. Lipid-bilayer nanodiscs are highly versatile in their compositions and surface charges, as they can be encapsulated by amphiphiles as diverse as membrane-scaffold proteins (MSPs), saponins, peptides, and amphiphilic copolymers. Such nanodiscs are primarily used for stabilizing membrane proteins within a native-like lipid-bilayer environment, yet under well-defined in vitro conditions. Due to their small size and large specific surface area compared to spherical nanoparticles, nanodiscs are also being investigated as carrier systems for small molecules, peptides, genes, or vaccines [11].

Through selective composition, surface functionalization, or loading of antigens, nanodiscs can selectively activate immune cells to achieve an immune response. For example, in a study by Noh et al., nanodiscs were developed for antibacterial vaccination. The lipid component of these nanodiscs consisted of a bacterial outer membrane encapsulated by a styrene–maleic acid (SMA) copolymer. Subsequent in vitro investigations in a DC2.4 mouse cell line and in vivo studies in mice showed increased DC maturation, efficient lymph node accumulation, and promotion of Th1- and Th17-directed cellular immunity, resulting in an effective immune response against the targeted bacteria [12]. Bhattacharya et al. employed a different approach by integrating recombinant hemagglutinin into nanodiscs for the treatment of influenza infection. This resulted in an increased adaptive (enhanced T-cell proliferation) and humoral (elevated IgM and IgG release) immune response in mice compared to hemagglutinin alone [13]. Another approach involved the use of high-density lipoprotein (HDL) nanodiscs, which interacted with cellular cholesterol and facilitated enhanced receptor-mediated uptake. In a study by Kuai et al., HDL nanodiscs were modified with a Toll-like receptor 9 (TLR9) agonist (i.e., 5′-C-phosphate-G-3′) attached to cholesterol, with the addition of tumor antigen peptides. This resulted in enhanced antigen–adjuvant co-delivery to the draining lymph nodes, prolonged antigen presentation by DCs, and a widespread T-cell response that led to the inhibition of tumor growth (including both CD4+ and CD8+ T cells) [14]. Subsequent advancements in this formulation were achieved by incorporating two distinct adjuvants (i.e., TLR4 and TLR9 agonists) and protein antigens, resulting in robust anti-tumor efficacy in multiple murine tumor models [15]. Apart from surface receptors such as TLRs, intracellular pathways of the innate immune system, e.g., stimulator of interferon genes (STING), can also be activated by agonists and trigger a robust immune response. Stimulation of this intracellular receptor induces the release of cytokines and chemokines, which in turn attracts immune cells and stimulates DC maturation and antigen presentation, which is associated with T-cell differentiation [16]. This principle was utilized by Dane et al., who conjugated cyclic dinucleotides (STING agonists) to PEGylated lipids in nanodiscs. The nanodiscs accumulated in tumor tissue models and led to activation of the STING pathway in tumor cells and colocalized DCs, resulting in up to 80% tumor rejection in a colorectal tumor model [17].

The studies described above demonstrate the considerable potential of nanodiscs in the field of immunotherapy. Most experiments utilize immortalized dendritic mouse cell lines. However, it has been observed that mouse models do not fully reflect what happens in vivo in humans, which can lead to discrepancies in immune responses and possibly inaccurate results [18]. Similarly, the immunomodulatory properties of NDDSs extend beyond their effect on DCs to other immune cells such as macrophages [19]. Consequently, a comprehensive understanding of the interactions between nanodiscs and human macrophages and DCs is imperative to accurately delineate their function in pathogen recognition, antigen presentation, and immune activation.

Considering this, the aim of this study was to investigate the immunomodulatory effects of two differently functionalized nanodisc formulations on human macrophages and DCs. To this end, a monocytic cell line (THP-1) was differentiated into resting macrophages (M0) and immature dendritic-like cells (iDCs) and carefully characterized with respect to morphology, stiffness, and surface marker expression. Following these studies, lipid-bilayer nanodiscs encapsulated by either an anionic copolymer glyco-DIBMA (diisobutylene–maleic acid) or an electroneutral polymer sulfo-DIBMA were examined for their potential application in immunotherapeutic contexts [20,21,22,23]. Biocompatible concentrations were applied to M0 and iDCs and reactive oxygen species (ROS) production, and cytokine release was analyzed. Furthermore, we investigated cell internalization of the nanodiscs and their co-localization with lysosomes, the endoplasmic reticulum (ER), and mitochondria. Finally, we determined cell activation by analyzing cell-specific surface marker expression in M0 and iDCs (i.e., CD80, CD86, human leukocyte antigen DR isotype (HLA-DR)).

## 2. Results

### 2.1. Differentiation and Characterization of Monocytes

**Figure 1:**
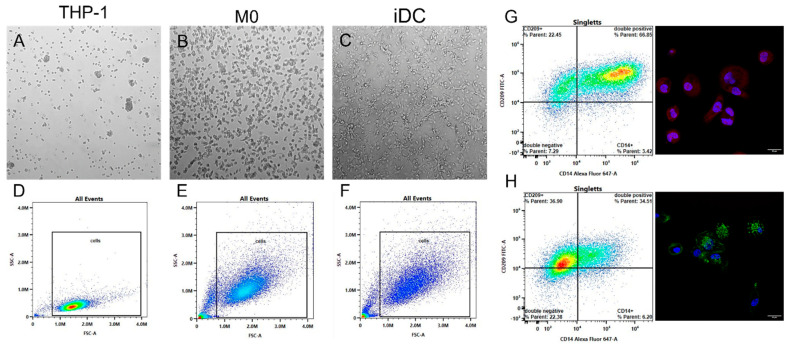
Morphology and flow cytometry investigations of THP-1 monocyte differentiation into M0 and iDCs. (**A**–**C**) Light microscopy images. (**D**–**F**) Flow cytometry plots (forward scatter (FSC-A(/side scatter (SSC-A)). M0 and immature dendritic-like cells (iDCs)show increased size and granularity relative to THP-1 cells. (**G**,**H**) Surface marker expression in M0 and iDCs: (**G**) flow cytometry plot and confocal microscopy image of M0, showing CD14+ expression indicated by the data plot, as well as the red CD14 signal in the microscopic image; scale bar = 20 µm (**H**) flow cytometry plot of iDCs indicating a CD209+ signal, confirmed by the green signal (CD209) in the confocal microscopy image; scale bar = 20 µm.

To examine the interactions of newly developed nanodiscs with human-derived immune cells, monocytes were differentiated and thoroughly characterized. The differentiation of monocytic THP-1 cells into M0 was successfully induced using 6.16 ng/mL PMA (phorbol-12-myristate-13-acetate). For iDCs, 20 ng/mL PMA and 20 ng/mL IL-4 (interleukin) were applied. Full differentiation was confirmed by light microscopy and flow cytometry measurements, as exemplified in Figure 1. The top-row microscopy images illustrate increased cell size and variations in shape in both M0 and iDCs compared to undifferentiated THP-1 cells (Figure 1A–C). Flow cytometry data (Figure 1D–F) confirmed these morphological changes, with forward scatter (FSC-A) and side scatter (SSC-A) profiles indicating enhanced cell size and granularity in both M0 and iDC populations. Figure 1G,H provide additional verification of cell phenotype through surface marker expression. In Figure 1G, M0 showed a strong CD14+ signal (Alexa Fluor 647), displayed as red staining in the confocal microscopy image, and a prominent CD14+/CD209+ quadrant in flow cytometry. In contrast, iDCs (Figure 1H) exhibited a lower CD14+ signal in parallel with prominent CD209 expression (FITC), marked by green fluorescence in the confocal image, and a CD209+ population in flow cytometry.

**Figure 2:**
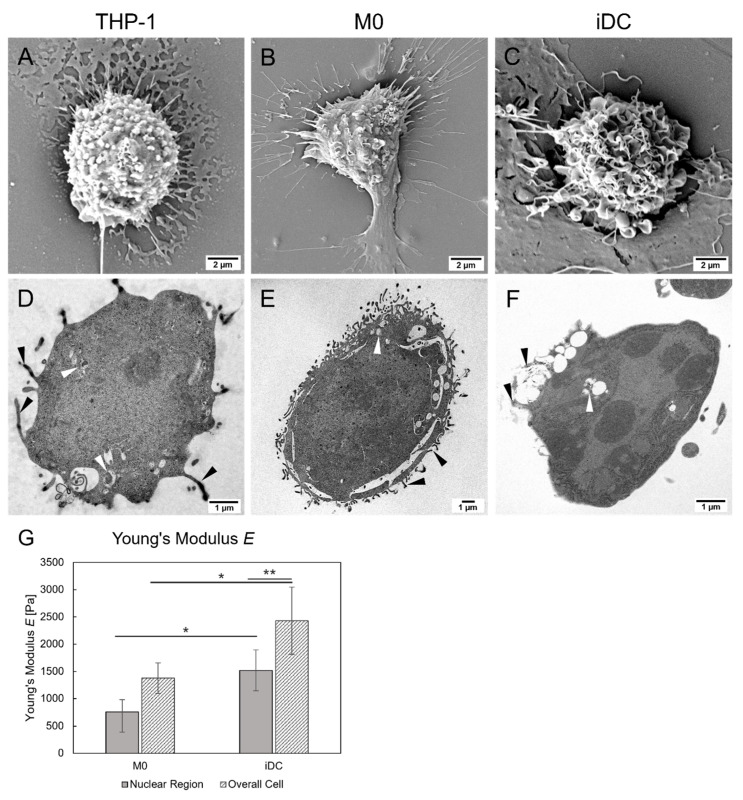
Morphological and mechanical characterization of immune cells. (**A**–**C**) Scanning electron icroscopy micrographs of THP-1 monocytes, M0, and iDCs reveal distinct cell sizes and surface morphologies. (**D**–**F**) Representative transmission electron microscopy (TEM) images showing inner organization (12,000× magnification), including phagosomes (white arrowhead) and cellular extrusions (black arrowheads) of the different cell types. (**G**) Young’s moduli E [Pa] of M0 and iDCs in the nuclear region and the overall cell show differences in cell mechanics, with overall higher values in iDCs. * *p* < 0.05, ** *p* < 0.01.

Scanning electron microscopy (SEM) and transmission electron microscopy (TEM) were performed to study surface morphology as well as inner cell organization (Figure 2). Figure 2A shows a THP-1 monocyte with smooth, spherical cell shape, minimal cellular extensions, and an average cell size of approximately 7 µm. In addition, no cell attachment was observed. By contrast, M0 and iDCs (Figure 2B,C) revealed morphological differences indicative of complete differentiation and an activated state. Both cell types exhibited characteristic cellular attachment, an increased cell size (~14 µm for M0; ~9 µm for iDCs), extended extracellular protrusions, and a ruffled cell surface, especially in iDCs. Regarding inner structure and cell organization a round cell shape (~7.7 µm), a large nucleus, and minimal formation of podosomes were found for THP-1 cells (Figure 2D). In comparison, the round-shaped M0 were larger (~16.6 µm), showing a typically kidney-shaped nucleus and increased podosome formation. Similar morphological features were found for iDCs, with an irregularly shaped cell nucleus and a round cell shape, exhibiting extrusions in form of podosomes. Additionally, phagosomes were identified in all three cell types, indicating phagocytic activity. Abundant cell organelles were found in every cell type.

The mechanical properties of M0 and iDCs were evaluated by assessing stiffness in both the nucleus and the overall cell area. The mean Young’s modulus *E* values (±SD) are presented in Figure 2G. In the nuclear regions, M0 exhibited significantly lower stiffness (762 ± 222 Pa) compared to iDCs (1519 ± 375 Pa), which showed a twofold increase in Young’s modulus *E* (*p* < 0.05). A similar pattern was observed for the overall cell stiffness, with a Young’s modulus *E* of 1376 ± 279 Pa for M0 and 2429 ± 618 Pa for iDCs (*p* < 0.05). In addition, stiffness was found to be lower in the nuclear regions compared to the overall cell in both cell lines, with a significant difference in iDCs (*p* < 0.01).

### 2.2. Nanoparticle–Immune Cell Interaction

#### 2.2.1. Nanoparticle Characterization

Glyco-DIBMA and sulfo-DIBMA nanodiscs were analyzed with regard to their particle size distribution and zeta-potential in phosphate buffer saline (PBS) and serum-free Roswell Park Memorial Institute (RPMI) 1640 medium after incubation for up to 24 h using dynamic light scattering (DLS). The hydrodynamic diameters (Table 1) obtained in PBS were 14.9 ± 0.3 nm and 13.2 ± 1.2 nm for glyco- and sulfo-DIBMA nanodiscs, respectively. Dilution and 24 h incubation in cell culture medium did not affect the size of the nanodiscs, confirming their stability, with results ranging from 11.4 nm to 17.7 nm for both formulations. The zeta-potentials of both nanodisc formulations were not affected by the medium used (PBS or RPMI 1640 medium) and remained stable over the tested time period (24 h). Glyco-DIBMA nanodiscs showed a zeta-potential between −12.6 mV and −15.5 mV, whereas the sulfo-DIBMA nanodiscs ranged from −2.0 mV to −3.5 mV. Overall, the nanodiscs formed stable dispersions with only minor, insignificant changes in size. This corroborates previous in-depth characterization of nanodiscs [20,22].

#### 2.2.2. Cytotoxicity

**Figure 3:**
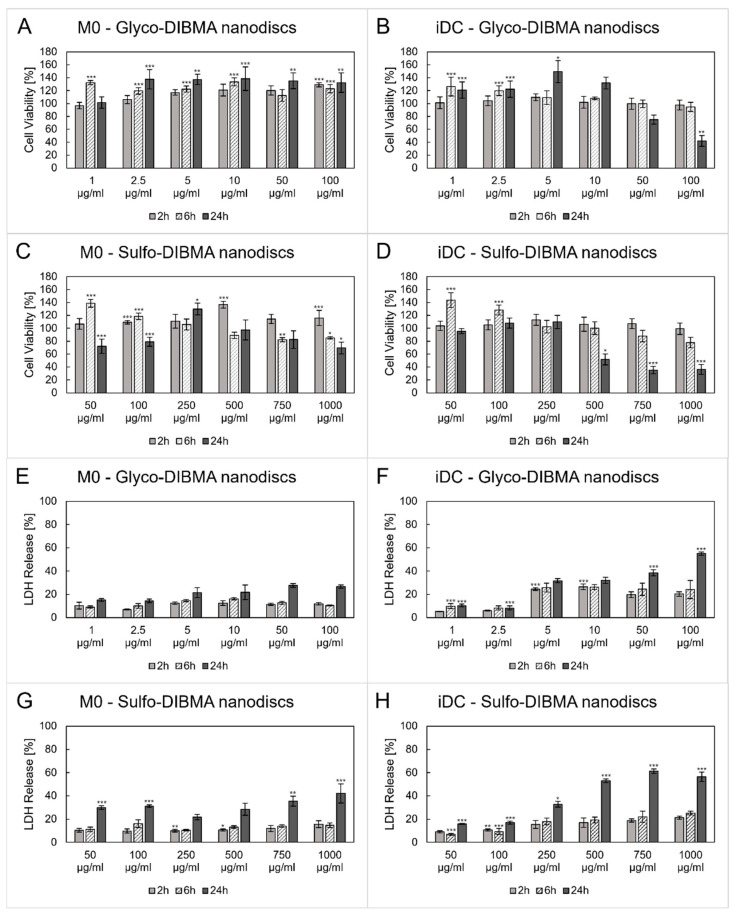
Cytotoxicity investigations of glyco- and sulfo-DIBMA nanodiscs in a time- (2, 6, 24 h) and concentration-dependent manner (1–100 µg/mL and 50–1000 µg/mL, respectively). (**A**–**D**) Higher concentrations led to a decrease in cell viability, while lower concentrations increased the mitochondrial activity in M0 and iDCs. (**E**–**H**) Significant lactate dehydrogenase (LDH) release was observed only in high concentrations after 24 h in M0 and iDCs. Samples were compared to an untreated cell control for statistical analysis. * *p* < 0.05, ** *p* < 0.01, *** *p* < 0.001.

The cytotoxic effects of glyco- and sulfo-DIBMA nanodiscs were evaluated in a concentration-dependent manner after 2, 6, and 24 h of incubation with differentiated M0 and iDCs. Based on preliminary studies, concentration ranges of 1–100 µg/mL for glyco-DIBMA and 50–1000 µg/mL for sulfo-DIBMA nanodiscs were selected for further analysis (Figure 3). The results showed a concentration- and time-dependent decrease in cell viability, reflected by reduced mitochondrial activity (Figure 3A–D) and an increase in lactate dehydrogenase (LDH) release, indicating comprised membrane integrity (Figure 3E–H). After 2 and 6 h of incubation, only minor cytotoxic effects on M0 and iDCs were observed. However, after 24 h, a significant decrease in cell viability (below 70%) was found for both cell types for the majority of the samples. In the case of sulfo-DIBMA nanodiscs, cytotoxic effects (i.e., LDH release >30%) on M0 were evident at the highest concentrations tested (i.e., 750 and 1000 µg/mL). The high LDH release of 35.5% ± 4.3% (*p* < 0.01) and 42.2% ± 8.2% (*p* < 0.001), respectively, implies significant cell membrane damage and consequent cell death [24]. In contrast, iDCs showed a significant decrease in cell viability already at 250 µg/mL, as indicated by an LDH release of 32.8% ± 2.5% (*p* < 0.05). A similar pattern of biocompatibility was found for glyco-DIBMA nanodiscs, but in a lower concentration range (i.e., 1–100 µg/mL). M0 displayed no cell membrane damage or impaired mitochondrial activity below 70% across the entire time and concentration range tested. In contrast, mitochondrial activity decreased to 74.9% ± 6.9% and 42.0% ± 8.4% (*p* < 0.01) at a concentration of 50 and 100 µg/mL in iDCs after 24 h, respectively. These results are consistent with the LDH release data, which were 38.6% ± 2.6% (*p* < 0.001) and 54.9% ± 1.4% (*p* < 0.001). Unlike the cytotoxic effects observed at higher concentrations, a significant increase in mitochondrial activity was seen at lower nanodisc concentrations. This increase was particularly evident after 6 and 24 h of incubation, independently of the formulation and cell line. The highest levels of mitochondrial activity were observed after 6 h of incubation with sulfo-DIBMA nanodiscs, resulting in a 1.4-fold increase in M0 and iDCs, and after 24 h of incubation with glyco-DIBMA nanodiscs, yielding a 1.4-fold upregulation in M0 and 1.5-fold increase in iDCs. As iDCs were found to be sensitive toward higher concentrations of nanodiscs, lower concentrations were used for subsequent experiments (i.e., 5 and 10 µg/mL for glyco-DIBMA nanodiscs; 50 and 100 µg/mL for sulfo-DIBMA nanodiscs).

#### 2.2.3. ROS Production

**Figure 4:**
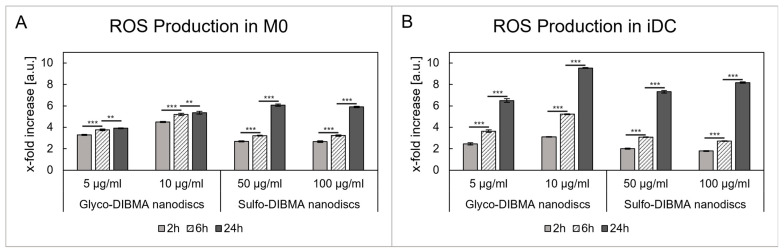
Nanodisc incubation leads to reactive oxygen species (ROS) generation in M0 (**A**) and iDCs (**B**). The results show a time-dependent increase, ranging from a 1.8-fold to 9.5-fold increase compared to an untreated cell control. ** *p* < 0.01, *** *p* < 0.001.

ROS generation was assessed by incubating M0 (Figure 4A) and iDCs (Figure 4B) with glyco- and sulfo-DIBMA nanodiscs for 2, 6, and 24 h. Both nanodisc formulations induced a time-dependent increase in ROS production in both cell types. Glyco-DIBMA nanodiscs led to a significant increase in ROS production in M0 after 2 h in both concentrations tested, which increased up to 3.9- and 5.4-fold after 24 h (*p* < 0.01) compared to an untreated cell control. Similarly, glyco-DIBMA nanodiscs induced a prominent increase in ROS production in iDCs, which also reached a peak after 24 h with a 6.5-fold and 9.5-fold increase (*p* < 0.001) at 5 and 10 µg/mL, respectively. For sulfo-DIBMA nanodiscs, ROS production in M0 increased more than 2-fold after 2 h and further to approximately 3.2-fold after 6 h at both tested concentrations (i.e., 50 and 100 µg/mL; *p* < 0.001). After 24 h, ROS levels were up to sixfold higher in M0. Similar trends were observed in iDCs, where ROS production increased in a time- and concentration-dependent manner, peaking at 24 h with a 7.3- and 8.2-fold increase in ROS production compared to an untreated cell control (*p* < 0.001).

#### 2.2.4. Cytokine Release

**Figure 5:**
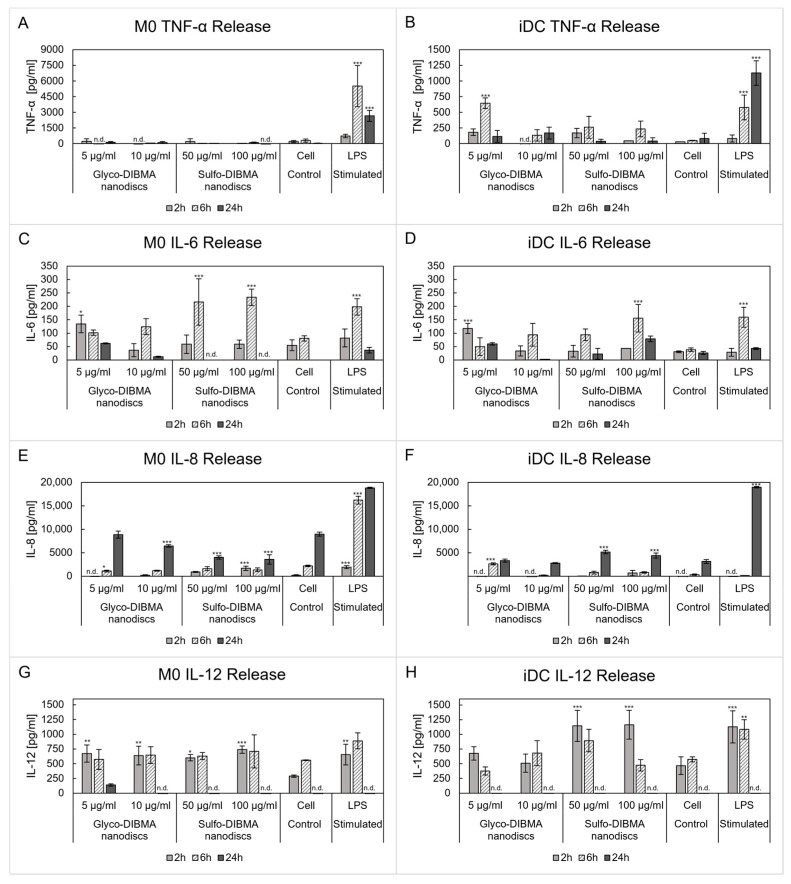
Cytokine release in M0 (**A**,**C**,**E**,**G**) and iDCs (**B**,**D**,**F**,**H**) induced by incubation with glyco-DIBMA and sulfo-DIBMA nanodiscs. Cell-specific variations in cytokine and chemokine release are observed. Untreated and lipopolysaccharide (LPS)-treated cells served as negative and positive cell controls. Samples were compared to an untreated cell control for statistical analysis. n.d., not detected * *p* < 0.05, ** *p* < 0.01, *** *p* < 0.001.

Cytokine release was analyzed to evaluate potential immunostimulatory effects of the nanodiscs. The pro-inflammatory cytokines tumor necrose factor (TNF)-α, IL-6, and IL-12, the anti-inflammatory cytokine IL-10, and the chemokine IL-8 were quantified (Figure 5). TNF-α production in M0 was detected exclusively in lipopolysaccharide (LPS)-stimulated cells, with a peak response observed after 6 h of incubation (i.e., 5520 ± 1969 pg/mL), indicating a robust pro-inflammatory response (*p* < 0.001). In iDCs, LPS stimulation induced a time-dependent increase in TNF-α release, but at lower levels than in M0. Glyco-DIBMA nanodiscs (5 µg/mL) induced LPS-like increases in TNF-α at 2 and 6 h (i.e., 183.9 ± 51.5 pg/mL and 654.0 ± 85.0 pg/mL, respectively; *p* < 0.001), whereas sulfo-DIBMA nanodiscs (50 and 100 µg/mL) caused a moderate increase compared to the cell control. After 24 h, TNF-α levels had dropped to basal levels in both M0 and iDCs. A distinct response was observed for IL-6 release, with a time-dependent response to LPS and nanodisc treatments that varied between cell types. In M0, both LPS and nanodisc treatments induced strong IL-6 responses at early time points (2 and 6 h). Sulfo-DIBMA nanodiscs at both tested concentrations significantly increased IL-6 release (up to 233.6 ± 30.6 pg/mL; *p* < 0.001), whereas glyco-DIBMA nanodiscs induced a weaker response (124.4 ± 29.8 pg/mL) at 6 h. A similar trend was observed in iDCs, with lower overall IL-6 levels compared to M0, yet significantly increased compared to the cell control.

IL-12 release was detected in both cell types after 2 and 6 h incubation. In M0, both nanodisc formulations induced IL-12 release at levels comparable to LPS-stimulated cells at 2 h, while IL-12 levels remain unchanged at 6 h (572.5–740.6 pg/mL). In iDCs, sulfo-DIBMA nanodiscs at both concentrations induced LPS-like IL-12 responses that decreased after 24 h, while glyco-DIBMA nanodiscs induced only a modest increase compared to controls. In addition to the cytokines, the chemokine IL-8, a potent chemoattractant of neutrophils, showed minimal induction by the nanodiscs. In M0, glyco-DIBMA nanodiscs produced slightly higher IL-8 release than sulfo-DIBMA nanodiscs, but levels remained within control ranges. No significant IL-8 induction was observed in iDCs, except for glyco-DIBMA nanodiscs at 6 h (2642 ± 210 pg/mL; *p* < 0.001) and sulfo-DIBMA nanodiscs at 24 h (5159 ± 372 pg/mL; *p* < 0.001), although these IL-8 levels remained in a low concentration range. In contrast, LPS strongly induced IL-8 release in both cell types, with the strongest responses at 6 h (M0) and 24 h (both M0 and iDCs). The anti-inflammatory cytokine IL-10 was not detectable, suggesting that neither formulation strongly promotes the release of anti-inflammatory cytokines.

#### 2.2.5. Uptake and Co-Localization Studies

**Figure 6:**
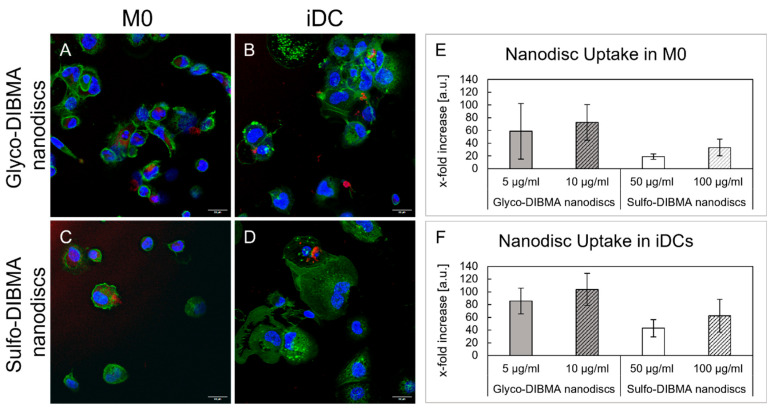
Successful nanodisc uptake of both formulations via active uptake pathways in M0 and iDCs after 24 h. (**A**–**D**) Microscopic images show glyco-DIBMA (10 µg/mL) and sulfo-DIBMA (100 µg/mL) nanodisc uptake indicated by the red signal (λ_Ex_ = 588 nm). Nuclei are shown in blue (λ_Ex_ = 405 nm) and cytoskeleton in green (λ_Ex_ = 488 nm). (**E**,**F**) X-fold increase in internalized nanodiscs compared to an untreated cell control. iDCs exhibited higher uptake in both formulations compared to M0; scale bar = 20 µm.

The uptake mechanisms (i.e., active or passive) of glyco-DIBMA and sulfo-DIBMA nanodiscs were investigated by microscopy and flow cytometry. We found that both formulations were internalized exclusively by active processes, as no nanodiscs were detected in samples assessing passive uptake at 4 °C. The red signal in Figure 6A–D indicates successful internalization of rhodamine-B labeled nanodiscs in M0 and iDCs. Notably, glyco-DIBMA nanodiscs were internalized to a greater extent than sulfo-DIBMA nanodiscs, as more cells exhibited visible nanodisc uptake. These observations were corroborated by flow cytometry analysis quantifying the mean rhodamine-B+ fluorescence intensity of cells (Figure 6E,F). Both nanodisc formulations showed concentration-dependent uptake, with glyco-DIBMA nanodiscs yielding overall higher mean fluorescence intensity (MFI) values in both cell types compared to an untreated cell control and sulfo-DIBMA nanodiscs treatment (Figure 6E). Moreover, nanodisc uptake was notably increased in iDCs compared to M0 for glyco-DIBMA and sulfo-DIBMA nanodiscs.

**Figure 7:**
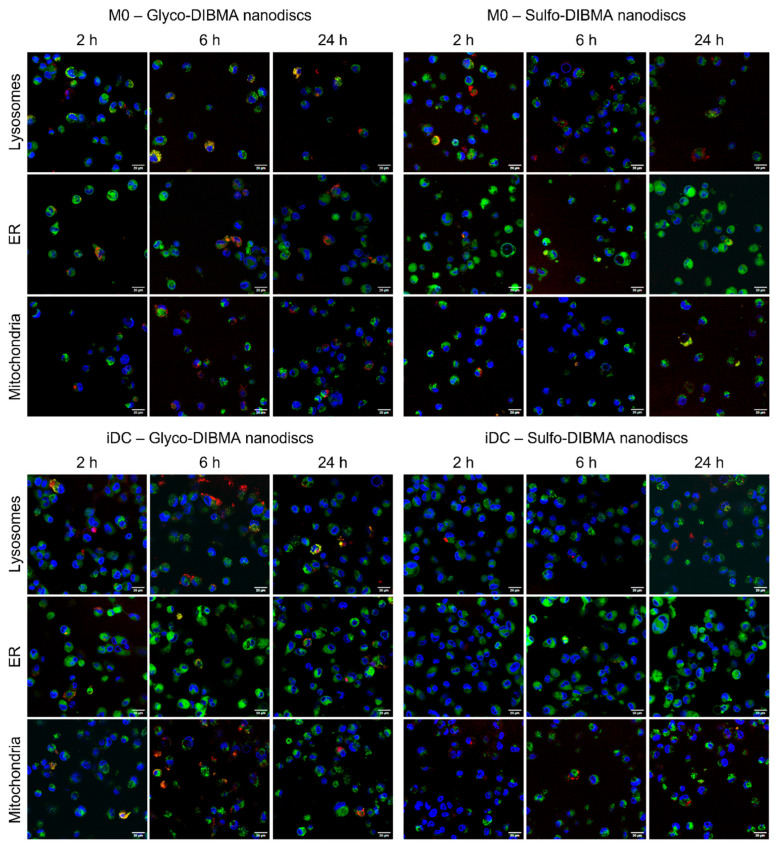
Live cell imaging of glyco- and sulfo-DIBMA nanodiscs colocalized with cell organelles, i.e., lysosomes, endoplasmic reticulum (ER), and mitochondria. Nuclei are shown in blue (λ_Ex_ = 405 nm), nanodiscs are shown in red (λ_Ex_ = 588 nm), and the cell organelles are visible in green (λ_Ex_ = 488 nm); scale bar = 20 µm.

Intracellular localization of glyco-DIBMA and sulfo-DIBMA nanodiscs was assessed over 24 h in M0 and iDCs, revealing distinct time-dependent distribution patterns (Figure 7). In M0 cells treated with glyco-DIBMA nanodiscs, initial localization at 2 h occurred near lysosomes and the ER, with minimal association with mitochondria. A similar pattern was observed at 6 h, while at 24 h, glyco-DIBMA nanodiscs were predominantly localized within lysosomes. In contrast, M0 cells treated with sulfo-DIBMALPs showed different behavior. At 2 h, nanodiscs were predominantly localized near lysosomes, with no detectable presence near the ER. At 6 h, colocalization with mitochondria and lysosomes increased, while the ER showed no association, which persisted at 24 h. In iDCs treated with glyco-DIBMA nanodiscs, nanodiscs were mainly localized near lysosomes, with a low presence near the ER at 2 h. At 6 h, the distribution shifted, with nanodiscs mainly near mitochondria and the ER, and minimal colocalization with lysosomes. At the last time point tested, nanodiscs were primarily localized in lysosomes, with some association near the ER and negligible presence near mitochondria. For iDCs treated with sulfo-DIBMA nanodiscs, nanodisc uptake was generally lower compared to glyco-DIBMA nanodiscs. At 2 h, nanodiscs were localized near lysosomes, with little or no association with the ER. This distribution persisted at 6 and 24 h, where nanodiscs were observed mainly near lysosomes and mitochondria with no colocalization with the ER.

#### 2.2.6. Immune Cell Activation

**Figure 8:**
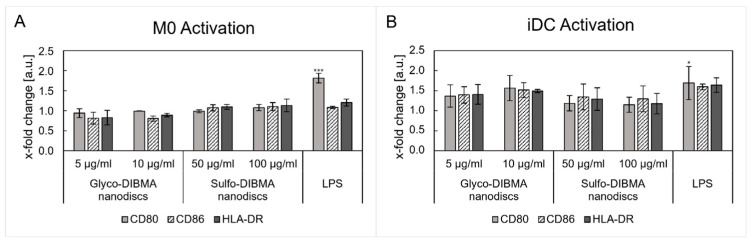
Surface marker expression of CD80, CD86, and human leukocyte antigen DR isotype (HLA-DR) on M0 (**A**) and iDCs (**B**) upon incubation with glyco- and sulfo-DIBMA nanodiscs. Sulfo-DIBMA nanodiscs did not alter surface marker expression in M0 or iDCs. Glyco-DIBMA nanodiscs induced a downregulation in M0 and an upregulation in surface marker expression and activation. Results are expressed as x-fold changes relative to an untreated cell control. Samples were compared to an untreated cell control for statistical analysis. * *p* < 0.05, *** *p* < 0.001.

The potential activation of immune cells was investigated by determining the expression of the co-stimulatory molecules CD80 and CD86 as well as HLA-DR, an MHC-II molecule essential for antigen presentation. Figure 8 displays the changes in surface marker expression after 24 h of nanodisc incubation in M0 and iDCs. LPS stimulation activated both cell types, resulting in an upregulation of the markers studied, showing a significant increase for CD80 (M0: *p* < 0.001; iDC: *p* < 0.05). However, nanodisc incubation in M0 did not induce cell activation. Glyco-DIBMA nanodiscs induced a downregulation in surface marker expression. In the case of sulfo-DIBMA nanodiscs, minimal effects on surface marker expression were observed, with expression levels comparable to the untreated control. Different results were found for iDCs. Glyco-DIBMA nanodiscs affected the expression of surface markers, resulting in approximately 1.4- and 1.5-fold increases in CD80, CD86, and HLA-DR expression at 5 and 10 µg/mL. Incubation with sulfo-DIBMA nanodiscs, on the other hand, did not alter surface marker expression, showing similar results to M0. Overall, the upregulation of surface markers in iDCs reflected their activation and maturation after glyco-DIBMA nanodisctreatment, whereas M0 were only slightly affected. Sulfo-DIBMA nanodiscs did not induce cell activation in either cell type.

## 3. Discussion

Immune cells are widely distributed throughout the body and act as a critical line of defense, strongly influencing the fate of drug-delivery vehicles. Upon administration, tissue-resident immune cells, like macrophages and DCs, recognize such drug-delivery vehicles and initiate either a pro- or anti-inflammatory response. This initial immune response, characterized by the production of ROS and the release of cytokines, activates cells (e.g., macrophages and DCs) and subsequently primes T-cell responses. The nature of this interaction determines whether the body mounts an immunogenic (pro-inflammatory) or tolerogenic (anti-inflammatory) response to drug-delivery vehicles, thereby shaping the body’s immune response.

Given the central role of immune cell interactions in determining NDDS responses, it is critical to identify in vitro models that accurately mimic immune responses. In our study, differentiation of human THP-1 cells was confirmed by the characteristic adherence, morphology, and surface marker expression unique to macrophages and DCs. Morphological analyses showed increased cell size and granularity in both M0 and iDCs, confirmed by microscopy and flow cytometry, with distinct shapes: M0 revealed a rounder morphology, while iDCs exhibited a dendritic and branched appearance, consistent with findings from Deng et al. [25]. Flow cytometry FSC/SSC plots and SEM images further highlighted increased granularity and surface irregularities in M0 and iDCs compared to the smooth surfaces of undifferentiated THP-1 monocytes, reflecting distinct activation states. The smoother monocyte surface supports their role as circulating precursors capable of migrating to inflammatory sites, where they differentiate into macrophages or DCs [26]. In contrast, the increased spreading and surface protrusions observed in M0 and iDCs are characteristic of adherent cells engaged in phagocytosis, as these cells form pseudopodia that facilitate adherence, migration, and particle ingestion [27,28]. Building on these surface and morphological changes, atomic force microscopy (AFM) analysis revealed lower stiffness in the nuclear regions of both M0 and iDCs, which aligns with findings by Labernadie et al., who reported lower stiffness in podosome-free areas of macrophages, such as nuclei [29]. This observation was further supported by Schimpel et al., who found increased elasticity in the nuclear regions of M cells due to sparse microvillus structures, indicating that F-actin organization modulates overall cell stiffness [30]. The 1.7-fold higher Young’s modulus *E* observed in iDCs likely reflects the formation of extracellular protrusions, as iDCs displayed higher expression of podosomes or dendritic projections than monocytes, linked to their active phagocytic and endocytic function. This uptake activity in iDCs corresponds with increased dendrite formation, which enables environmental sensing and uptake of external particles or antigens, supported by restructuring of F-actin filaments at the cell surface. In contrast, M0 exhibited fewer podosomes and lower F-actin abundance, consistent with their resting state and lower stiffness, as evident in the SEM images [31]. In addition to cell morphology, the expression of cell-specific surface markers is an integral part of successful differentiation. The expression of CD14, a PRR for LPS and a marker for phagocytosis on M0, confirms the monocyte–macrophage differentiation, as monocytes typically lack CD14 expression [32,33,34]. Additionally, M0 cells showed an overlapping CD209 expression, which is in accordance with data found in the literature [35]. On the contrary, iDCs mainly expressed the surface marker CD209 (i.e., DC-SIGN, dendritic cell-specific ICAM-3-grabbing non-integrin), indicating successful differentiation and functionality. The C-type lectin membrane-bound receptor is known for its role in intercellular adhesion, antigen uptake and presentation, and modulation of the immune response [36,37]. This suggests that iDCs and M0 are not only capable of receptor-mediated endocytosis and phagocytosis but also of T-cell signaling [32]. These results indicated that differentiation of THP-1 monocytes had been successfully achieved, as evidenced by the presence of distinct morphological features in M0 and iDCs.

Polymer-encapsulated lipid-bilayer nanodiscs are primarily used to investigate membrane proteins, but have substantial potential as NDDSs, as reported by Dong et al. [38]. The copolymer nanodiscs used in this study are based on the zwitterionic phospholipid DMPC encapsulated by either anionic glyco-DIBMA or electroneutral sulfo-DIBMA polymers. Both kinds of nanodiscs exhibited hydrodynamic diameters of about 14 nm and high stability, as reported previously [20,21]. Initial cytotoxicity evaluations indicated biocompatibility at lower concentrations, that is, between 5 and 100 µg/mL. However, it was found that M0 cells were less sensitive to nanodisc exposure compared to iDCs. This observation was consistent for both formulations, with sulfo-DIBMA nanodiscs showing higher tolerance at concentrations of 50–100 µg/mL compared to glyco-DIBMA nanodiscs, which were tolerated at 5–10 µg/mL. At higher concentrations, both formulations reduced cell viability in a dose-dependent manner. Notably, although the tested concentration range for glyco-DIBMA nanodiscs (1–100 µg/mL) was generally considered low, cytotoxic effects were already observed in iDCs at 50 and 100 µg/mL, indicating greater sensitivity of these cells compared to M0. Furthermore, M0 cells generally displayed greater tolerance; however, significant LDH release and reduced cell viability were evident after 24 h of exposure to 750 and 1000 µg/mL sulfo-DIBMA nanodiscs. This pronounced cytotoxic effect is likely due to membrane disruption at these high concentrations, as indicated by the increased LDH levels. Interestingly, at lower doses, mitochondrial activity increased up to 1.5-fold compared to untreated controls, which correlated with a significant increase in ROS production in both cell types [39]. M0 showed a lower maximum ROS increase of up to 5.3-fold than iDCs, in which ROS levels were elevated up to 9.5-fold over the tested period. Thus, glyco-DIBMA nanodiscs induced greater ROS production than sulfo-DIBMA nanodiscs despite their lower applied concentrations. This differing response may be attributed to the enhanced compatibility of nanodiscs with M0, as shown in the cytotoxicity results. Importantly, ROS production in immune cells serves purposes besides indicating cellular stress. As signaling molecules, ROS regulate processes such as cell growth, differentiation, and apoptosis and are also produced upon phagocytic and endocytic processes [40]. ROS have been reported to influence M0 polarization, supporting both the pro-inflammatory M1 phenotype and the transition to the anti-inflammatory M2 phenotype [41,42]. In DCs, ROS are critical for maturation and antigen presentation to T cells. The intracellular release of ROS has the potential to alkalinize the cytosolic environment, thereby reducing antigen degradation within phagosomes and promoting efficient antigen presentation on MHC class I and II molecules [43].

However, the release of ROS and the subsequent initiation of downstream processes can be triggered by cellular uptake of NDDSs, among other mechanisms. Microscopic investigations confirmed the successful internalization of nanodiscs by the cells. Specifically, glyco-DIBMA nanodiscs exhibited stronger fluorescence signals in M0 and iDCs compared to sulfo-DIBMA nanodiscs. Flow cytometry further corroborated these findings, revealing an approximately 1.3-fold increase in fluorescence signal in M0 and a 1.4-fold increase in iDCs for glyco-DIBMA compared to sulfo-DIBMA nanodiscs. This higher uptake efficiency of glyco-DIBMA nanodiscs aligns with their observed cytotoxicity and ROS production at biocompatible concentrations. The differences in uptake efficiency between glyco-DIBMA and sulfo-DIBMA nanodiscs is most likely due to differences in the surface charge between the two nanodisc formulations. Negatively charged nanoparticles, like glyco-DIBMA nanodiscs, are known to preferentially interact with phagocytes due to their similarity to microbial surface charges, thereby facilitating uptake [44].

The distinct uptake efficiencies of glyco- and sulfo-DIBMA nanodiscs suggest different endocytic pathways, as passive diffusion was excluded for both formulations and cell types. Colocalization studies highlighted divergent intracellular dynamics: glyco-DIBMA nanodiscs followed a time-dependent lysosomal processing pathway. In M0, these nanodiscs were initially localized near lysosomes and the ER, progressing to lysosomal accumulation within 24 h. In iDCs, glyco-DIBMA nanodiscs showed a broader distribution, with early lysosomal proximity transitioning to interactions with mitochondria and the ER at 6 h, and predominantly lysosomal localization at 24 h, possibly reflecting antigen-presenting processes. Conversely, sulfo-DIBMA nanodiscs exhibited lower uptake efficiency and restricted localization, with increasing colocalization to lysosomes and mitochondria in both cell types, but minimal ER interaction, implying different endocytic mechanisms.

In addition, the different surface charges of the nanodiscs likely influenced their uptake mechanisms. Phagocytic cells such as macrophages and DCs utilize PRRs, including TLRs, scavenger receptors (SRs), and C-type lectin receptors (CLRs), to internalize NDDSs [45]. The receptors differ in their ability to recognize specific surface patterns; for instance, TLR4 binds LPS, whereas SRs recognize lipoproteins. In the context of glyco-DIBMA nanodiscs, CLRs play a particularly important role, as these receptors are specialized in recognizing and binding to carbohydrate and sugar structures. Notable examples of such receptors include DC-SIGN, which is highly expressed on iDCs, the mannose receptor CD206, and dectin 1 [46]. The N-methyl-D-glucamine (“meglumine”) moiety on glyco-DIBMA may enhance binding to CLRs, thereby facilitating cellular internalization. In contrast, sulfo-DIBMA nanodiscs are likely internalized through less specific pathways, such as clathrin- or caveolin-mediated endocytosis, which do not require receptor-specific binding [47]. These findings likely contribute to the inertness and reduced uptake of sulfo-DIBMA nanodiscs [48]. However, the specific uptake mechanisms need to be investigated in more detail with the use of inhibitors of the specific uptake pathways.

NDDS uptake initiates various intracellular processes. As phagocytic cells, M0 and DCs play roles in modulating immune responses via cytokine secretion. Cytokines are pivotal in directing pro-inflammatory or anti-inflammatory responses and influencing cell activation, polarization, and maturation [7,8]. Analysis of pro-inflammatory (TNF-α, IL-12), anti-inflammatory (IL-10), and immunomodulatory (IL-6) cytokines and chemokines (IL-8) revealed distinct immune responses to glyco-DIBMA and sulfo-DIBMA nanodiscs, with formulation- and cell type-dependent differences. In contrast, LPS treatment served as control and consistently induced strong cytokine release. Measuring TNF-α levels revealed low levels for both nanodisc formulations. Only at the earlier test points did both types of nanodiscs elicit mild TNF-α increases, particularly in iDCs, indicating a moderate ability to activate these cells [8,49]. Similarly, IL-8 release was minimal, demonstrating the nanodiscs’ limited role in inducing chemotactic signals. Differences in cytokine release were observed for IL-6 and IL-12 release. Here, glyco-DIBMA nanodiscs demonstrated lower immune activation potential than sulfo-DIBMA nanodiscs, showing lower or comparable cytokine release. In contrast, sulfo-DIBMA nanodiscs exhibited higher pro-inflammatory potential, particularly through increased IL-6 production in M0 and stronger IL-12 induction in iDCs at early time points. This IL-12 release is also an explanation for the lack of IL-10 release, as these two cytokines are reported to be negative regulators [50]. However, the cytokine responses were transient, indicating a restricted potential for sustained immune activation [49,51]. In terms of maturation activation of iDCs, the data indicate a moderate pro-inflammatory response. The late decrease in IL-12 after 24 h indicates potential iDC maturation, as reported by Ebner et al. [52]. Accordingly, IL-12 is released during iDC maturation but decreases as maturation progresses and reaches a terminal phase. Similar results were found by Han et al., who reported a decrease in IL-12 release after 8 h of maturation induction using LPS and interferon (IFN)-γ [53].

The maturation and activation of DCs and M0 in general is characterized by the expression of specific surface markers, such as CD80, CD86, and HLA-DR [8,54]. These markers are not only associated with activation, maturation, and M1-like polarization but also with antigen presentation, which is essential for APC–T-cell interactions. Surface marker expression analyses elucidated cellular activation upon nanodisc incubation. Neither glyco- nor sulfo-DIBMA nanodiscs upregulated CD80, CD86, or HLA-DR in M0, indicating minimal activation or polarization, coinciding with the low cytokine release [54]. In contrast, significant upregulation of these markers was observed in iDCs, particularly with glyco-DIBMA nanodiscs. This upregulation suggests enhanced maturation and antigen-presenting capacity driven by glyco-DIBMA nanodiscs. Sulfo-DIBMA nanodiscs did not induce changes in expression levels. These differences in the formulations align with the higher uptake efficiency of glyco-DIBMA nanodiscs, likely mediated by specific PRR interactions, which not only result in higher uptake efficiency but also in increased maturation and antigen presentation [55]. Supporting this hypothesis, increased CD86 expression has been linked to higher nanoparticle uptake in previous studies, emphasizing the importance of receptor-mediated mechanisms in modulating immune responses [56].

Finally, the elevated expression of the co-stimulatory molecules CD80 and CD86 and the MHC-II protein HLA-DR may indicate the potential for T-cell activation and initiation of an adaptive immune response in vivo. The simultaneous binding of CD80–CD86 to the corresponding receptors on T cells, i.e., CD28 or CTLA-4, and peptide-MHC-II complexes to specific TCR molecules, promotes the activation and differentiation of CD4+ T cells into Th cells [57,58]. The cytokine release associated with iDC activation after incubation with glyco-DIBMA nanodiscs suggests a potential for Th17 or Th1 differentiation in vivo that could be mediated by IL-6 (Th17) and IL-12 release (Th10), observed in this in vitro system [59]. Furthermore, the activation of B cells through MHC-II presentation and co-stimulation could represent a valuable mechanism for enhancing immune responses, highlighting its potential relevance for vaccine development and further investigation [60]. Future studies will elucidate the precise mechanism(s) by which glyco-DIBMA nanodiscs could influence these pathways.

## 4. Materials and Methods

### 4.1. Materials

Dulbecco’s modified Eagle’s medium (DMEM), RPMI 1640 medium, RPMI 1640 medium without phenol red, PBS (pH 7.4), fetal bovine serum (FBS), penicillin–streptomycin (PEST), Hank’s balanced salt solution (HBSS), 0.25% trypsin–ethylenediaminetetraacetic acid (trypsin–EDTA) and recombinant human IL-4 were purchased from Gibco, Life Technologies Corporation (Painsley, UK). PMA, hydrogen peroxide (H_2_O_2_), minimum essential medium (MEM) nonessential amino acid solution (100×; NEAA), bovine serum albumin (BSA), 10% neutral buffered formalin solution, ionomycin, EDTA and BioTracker 488 green mitochondria dye were obtained from Sigma Aldrich (Munich, Germany). Alexa Fluor^®^ 647 anti-human CD14 antibody, fluorescein-5-isothiocyanate (FITC) anti-human CD209 (DC-SIGN) antibody, brilliant violet 650 anti-human CD80 antibody, PE–cyanine5 anti-human CD86 antibody, and APC–cyanine7 anti-human HLA-DR antibody were purchased from BioLegend (San Diego, CA, USA). Alexa Fluor 488 phalloidin, Hoechst 33342, ER-Tracker™ green dye, LysoTracker green DND-26, TNF-α recombinant human protein and dihydroethidium (DHE) were obtained from Thermo Fisher Scientific (Vienna, Austria). Ultra-purified water (i.e., Milli-Q water (MQ-water); Millipore SAS, Molsheim, France) was used for all experiments.

### 4.2. Cell Culture and Differentiation

The human acute monocytic leukemia cell line THP-1 was kindly provided by E. Fröhlich (Medical University of Graz, Austria). The cells were cultivated in RPMI 1640 medium supplemented with 10% FBS, 1% PEST, and 1% NEAA at 37 °C and a water-saturated atmosphere (5% CO_2_). Sub-cultivation was performed twice a week. THP-1 cells were differentiated into M0 and iDCs. To induce differentiation into M0 cells, THP-1 cells were cultured for 72 h in RPMI 1640 medium containing 6.16 ng/mL PMA. The medium was then changed to the standard RPMI 1640 medium and the cells were cultured for an additional 24 h for a resting phase. Differentiation into iDCs was performed by incubating the cells in RPMI 1640 medium supplemented with 20 ng/mL PMA and 20 ng/mL IL-4. After 96 h, the differentiation medium was changed back to RPMI 1640 medium and the cells were used for further experiments. In addition, the morphology of the differentiated cells was examined using a light microscope (Leica DM IL, Leica Microsystems GmbH, Wetzlar, Germany).

### 4.3. Cell Characterization

#### 4.3.1. Fluorescence Microscopy

After differentiation, M0 and iDCs express different surface markers, namely CD14 and CD209, respectively. To confirm the successful expression of the surface markers, fluorescence microscopy was performed. For this purpose, THP-1 cells (7.5 × 10^4^ cells/well) were seeded in 8-well chamber slides (Lab-Tek II RS Glass, Thermo Fisher Scientific, Vienna, Austria) and differentiated into M0 and iDCs. After this, the cells were fixed, permeabilized, and blocked according to the manufacturer’s protocol. The cell surface markers were then stained with Alexa Fluor 647 anti-human CD14 (M0) and FITC anti-human CD209 antibodies (iDCs) diluted in 0.1% BSA (1.5:200, *v*/*v*) for 3 h at room temperature. After three wash cycles, the nuclei were counterstained with Hoechst 33342 (24 µg/mL) and incubated for 25 min at room temperature. Finally, the cells were thoroughly washed again before the slides were mounted and visualized with a confocal laser scanning microscope (cLSM) using a Stellaris 5 confocal microscope (Leica) equipped with an LAS X 3.10.0 software package.

#### 4.3.2. Spectral Flow Cytometry

For further characterization of the differentiated cells regarding their size and granularity as well as the expression of cell-specific surface markers, flow cytometry was used. THP-1 cells (7.5 × 10^5^ cells/well) were seeded in a 24-well plate (Cellstar, Greiner Bio-One GmbH, Friedrichshafen, Germany) and differentiated into M0 and iDCs, as described above. Cells were harvested with 0.25% trypsin–EDTA, centrifuged at 450× *g* for 5 min and resuspended in a 2% BSA solution for 1 h to block non-specific staining. Surface markers, i.e., Alexa Fluor 647 anti-human CD14 (M0) and FITC anti-human CD209 antibody (iDCs), were then added and incubated at 4 °C for a further 30 min. After a subsequent wash and centrifugation step, the mean fluorescence intensity (MFI) was measured using Cytek Aurora (Cytek Biosciences, Fremont, CA, USA) and Cytek SpectroFlow^®^ 3.3.0 software.

#### 4.3.3. Atomic Force Microscopy

Cells exhibit different mechanical and elastic properties based on their function, healthy or diseased state, and environment [61]. To determine the cell mechanics of differentiated M0 and iDCs, AFM measurements were performed. For this purpose, the Young’s modulus *E* (Pa) of the entire cell surface and the nuclear region was investigated. THP-1 cells (1 × 10^5^ cells/well) were seeded on glass slides in Willco dishes (50/40, WillCo Wells B.V., Amsterdam, The Netherlands) and differentiated into M0 and iDCs according to the abovementioned protocol. Cells were observed in a liquid environment with a soft contact cantilever qpSCont (NanoAndMore GmbH, Wetzlar, Germany), which has a resonant frequency of 11 kHz and a spring constant of 0.01 N/m. A force of 200 pN and a velocity of 5 μm/s were used for the measurements. Prior to each experimental day, a new cantilever was calibrated to determine the spring constant using the software-integrated Sadder method. The deflection sensitivity of each cantilever was determined with a calibration under experimental liquid condition on a cover glass as a background and calculated in the AFM software (version 3.10.0.23) (C3000—Nanosurf). The data were acquired using single force curves with grids of 30 µm × 30 µm across the cells. All grids were positioned to capture the background and the center of the cell. For the analysis of the stiffness or cell elasticity of the cells, Young’s modulus *E* was calculated in the SPIP software (version 6.4.4.) using the Sneddon indentation model and a Poisson’s ratio of 0.5.

#### 4.3.4. Scanning and Transmission Electron Microscopy

THP-1 cells, M0 cells, and iDCs were visualized using SEM and TEM. For this, THP-1 cells (5 × 10^5^ cells/well) were seeded and grown on a poly-L-lysine (0.01%, Sigma Aldrich, Munich, Germany)-coated Aclar film and differentiated into M0 and iDCs. To obtain images via SEM, the cells were fixed for 30 min (2% paraformaldehyde and 2.5% glutaraldehyde in 0.1 M cacodylate buffer at pH 7.4) and washed for a further 30 min in 0.1 M cacodylate buffer (pH 7.4). The samples were then dehydrated (ethanol series 30%, 50%, 70%, 80%, 90%, and 96%, each for 15 min), rinsed twice for 5 min with absolute ethanol, and transferred to pure acetone. This was followed by drying with a BalTec CPD 030 critical point dryer (Balzers, Liechtenstein) and sputter-coating with a BalTec SCD 500 sputter coater (Balzers, Liechtenstein). Imaging was performed with a Zeiss Sigma 500 VP scanning electron microscope (Zeiss, Oberkochen, Germany) at a magnification of 1900, which operated at 5 kV with an Everhart–Thornley detector for secondary electrons. The Zeiss SmartSEM V05.04 imaging software was used for image acquisition.

For TEM, THP-1, M0 and iDCs were first fixed in 2.5% (*w*/*v*) glutaraldehyde and 2% (*w/v*) paraformaldehyde in 0.1 M cacodylate buffer (pH 7.4) for 1 h, followed by an additional fixation step with 2% (*w*/*v*) osmium tetroxide. Samples were then dehydrated in a gradual ethanol series (50%, 70%, 90%, 96%, and 100%, each for 30 min) and bedded in TAAB epoxy resin (Agar Scientific Ltd., Essex, UK). Ultrathin sections (75 nm) were prepared using a Leica UC7 ultramicrotome (Leica, Vienna, Austria) and placed on copper grids for imaging. Samples were examined at 120 kV using a Tecnai G2 FEI (Thermo Fisher Scientific, Vienna, Austria) microscope equipped with a Gatan Ultrascan 1000 CCD camera at 12,000× magnification.

### 4.4. Nanodisc Interaction Studies

#### 4.4.1. Nanodisc Preparation

**Figure 9:**
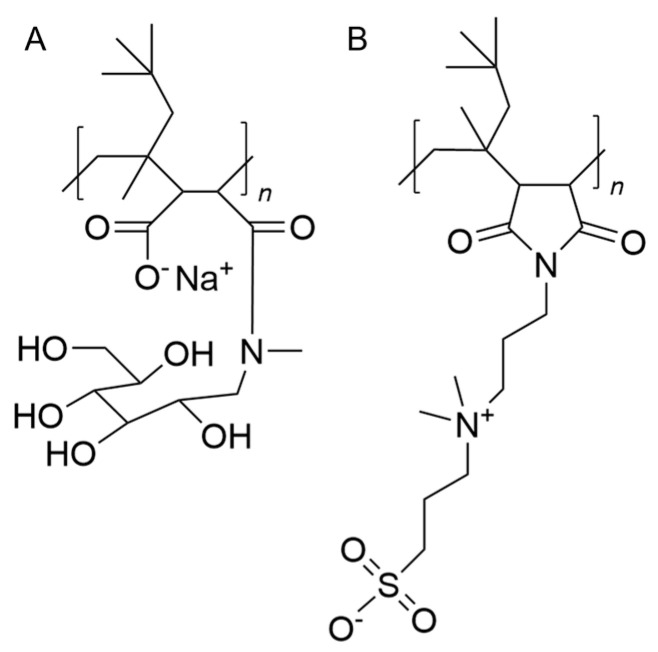
Chemical structure of (**A**) glyco-DIBMA and (**B**) sulfo-DIBMA polymers used for the nanodisc preparation.

Glyco- and sulfo-DIBMA nanodiscs were prepared according to Danielczak et al. (2022) and Glueck et al. (2022). The respective polymer structures are illustrated in Figure 9 [20,22]. Briefly, DMPC (1,2-dimyristoyl-*sn*-glycero-3-phosphocholine) powder was resuspended in PBS (*c*_DMPC_ = 20 mg/mL), briefly vortexed and then incubated at 35 °C and 500 rpm for 1 h. The DMPC multilamellar vesicles were then solubilized overnight in glyco- or sulfo-DIBMA to a final DMPC concentration of 10 mg/mL (35 °C, 500 rpm). For visualization of the nanodiscs, fluorescence-labeled DMPC was used (1 mol% rhodamine-PE). DLS measurements were then performed to confirm the successful preparation and stability. For this, the freshly prepared nanodiscs were diluted with PBS or RPMI 1640 medium (5 mg/mL) and directly measured. Additional measurements were performed after 24 h incubation in RPMI 1640 medium to simulate cell interaction study conditions. Size determination was performed using a Zetasizer (Nano ZS, Malvern Instruments, Worcestershire, UK) at 37 °C and a scattering angle of 173°, considering the respective predefined refractive indices (i.e., 1.330 PBS, 1.334 RPMI 1640 medium, 1.337 glyco-DIBMA, 1.338 sulfo-DIBMA). Zeta-potential measurements were conducted via laser Doppler–microelectrophoresis coupled with DLS. For this, samples were diluted as described above and measurements were performed at 37 °C applying a scattering angle of 173°.

#### 4.4.2. Cytotoxicity

To evaluate possible cytotoxic effects of the nanodiscs on the cells, 5 × 10^4^ THP-1 cells/well were differentiated into M0 and iDCs in 96-well plates (Cellstar, Greiner Bio-One GmbH, Friedrichshafen, Germany). This was followed by 2-, 6-, and 24 h incubation with glyco-DIBMA nanodiscs (1–100 µg/mL) and sulfo-DIBMA nanodiscs (50–1000 µg/mL). Cell viability was tested by applying an MTS assay (CellTiter 96 aqueous nonradioactive cell proliferation assay, Promega, Madison, WI, USA) according to the manufacturer’s protocols. After 2 h incubation of the substrate solution, absorbance was measured at 490 nm using a UV-vis plate reader (CLARIOstar^Plus^, BMG LABTECH, Ortenberg, Germany). Untreated cells represented 100% viability. In addition to this, cell membrane integrity was investigated by measuring LDH release using a CytoTox-ONE homogeneous membrane integrity assay (Promega, Madison, WI, USA). Following the manufacturer’s instructions, fluorescence was measured with a UV-vis plate reader (CLARIOstar^Plus^, BMG LABTECH, Ortenberg, Germany) at *λ*_Ex_ = 560 nm and *λ*_Em_ = 590 nm after 25 min incubation at 37 °C. Lysed cells (2% lysis solution) were used as 100% LDH release controls. All results were blank-corrected.

#### 4.4.3. Generation of ROS

ROS production was investigated using DHE. THP-1 cells at a density of 7.5 × 10^4^ cells/well were seeded and differentiated into M0 and iDCs in a 96-well plate (Sensoplate, Greiner Bio-One GmbH, Friedrichshafen, Germany). After washing with PBS, glyco-DIBMA nanodiscs (5 and 10 µg/mL) and sulfo-DIBMA nanodiscs (50 and 100 µg/mL) were added to the cells and incubated for 2, 6, and 24 h at 37 °C. Afterward, the cells were washed twice with PBS and incubated with DHE (10 µM) for 1 h. Fluorescence was then measured at *λ*_Ex_ = 518 nm and *λ*_Em_ = 612 nm using a UV-vis plate reader (CLARIOstar^Plus^, BMG LABTECH, Ortenberg, Germany). Untreated cells were used as a cell control. All results were blank-corrected.

#### 4.4.4. Cytokine Production—ELISA

In order to quantify the production of pro- and anti-inflammatory cytokines and chemokines (i.e., TNF-α, IL-6, IL-8, IL-10, IL-12), enzyme-linked immunosorbent assays (ELISA) were performed. THP-1 cells (7.5 × 10^5^) were seeded in 96-well plates (Cellstar, Greiner Bio-One GmbH, Friedrichshafen, Germany) and differentiated into M0 and iDCs as described above. Cells were washed with PBS prior to adding glyco-DIBMA nanodiscs (5 and 10 µg/mL) and sulfo-DIBMA nanodiscs (50 and 100 µg/mL) to the cells and incubated for 2, 6, and 24 h. Subsequently, supernatants were collected and centrifuged to remove any particulate substances and used for the following ELISA assays. To quantify cytokine and chemokine release, a human IL-6, IL-8, IL-10, and IL-12 ELISA set (BD Biosciences, San Jose, CA, USA) and human TNF-α ELISA kit (Thermo Fisher Scientific, Vienna, Austria) were applied according to the manufacturer’s instructions. Absorbance was measured at 450 nm using a UV-vis plate reader (CLARIOstar^Plus^, BMG LABTECH, Ortenberg, Germany). Untreated cells as well as cells treated with 1 µg/mL LPS were used as control. All results were blank-corrected.

#### 4.4.5. Uptake and Co-Localization Studies

To determine the internalization pathways of the nanodiscs, uptake studies with regard to uptake pathway and co-localization were performed. THP-1 cells (1.5 × 10^5^ cells/well) were seeded in 8-well chamber slides (Falcon, Corning, Glendale, AZ, USA) and differentiated into M0 and iDCs. First, it was assessed whether the nanodiscs were internalized via passive or active transport mechanisms. For this, the cells were incubated with rhodamine-labeled glyco- (10 µg/mL) and sulfo-DIBMA nanodiscs (100 µg/mL) for 4 h at 4 °C and 37 °C. At 4 °C, active transport processes were inhibited, allowing the assessment of passive uptake, while uptake at 37 °C included both passive and active transport mechanisms. Untreated cells were treated identically and used as cell controls. Following the nanodisc incubation, cells were fixed, permeabilized, and stained using Hoechst 33342 and Alexa Fluor 488 phalloidin to visualize the cell nuclei and cytoskeleton, respectively.

To further quantify nanodisc uptake, differentiated M0 and iDCs (1 × 10^6^ cells/well) were incubated with both nanodisc formulations for 24 h at 37 °C (5 and 10 µg/mL glyco-DIBMA nanodiscs, 50 and 100 µg/mL sulfo-DIBMA nanodiscs). After this, cells were washed with PBS and detached using 0.02% EDTA (in PBS). After a centrifugation step, the MFI of the rhodamine-labeled nanodiscs was measured with a Cytek Aurora (Cytek Biosciences, Fremont, CA, USA) equipped with Cytek SpectroFlow^®^ 3.3.0 software.

Next, co-localization studies were performed. For this, 1.5 × 10^5^ THP-1 cells/well were seeded and differentiated in 96-well plates (Sensoplate, Greiner Bio-One GmbH, Friedrichshafen, Germany). After complete differentiation, nanodiscs were added and incubated for 2, 6, and 24 h (10 µg/mL glyco-DIBMA nanodiscs, 100 µg/mL sulfo-DIBMA nanodiscs), followed by staining of the lysosomes, ER, and mitochondria. For this, 100 nM LysoTracker green DND-26 (Thermo Fisher Scientific, Vienna, Austria), 1 µM ER-Tracker green (Thermo Fisher Scientific, Vienna, Austria) and 200 nM BioTracker 488 green mitochondria dye (Sigma Aldrich, Munich, Germany) were used following the manufacturer’s protocols. Cell nuclei were counterstained with Hoechst 33342 (24 µg/mL) and visualization was performed using cLSM.

#### 4.4.6. Activation of M0 and iDCs

Flow cytometry was used to investigate the possible activation of immune cells by detecting cellular surface markers, i.e., co-stimulatory molecules CD80 and CD86 as well as the antigen-presenting HLA-DR. For this, M0 and iDCs were incubated with glyco- and sulfo-DIBMA nanodiscs (5 and 10 µg/mL and 50 and 100 µg/mL, respectively) or LPS (1 µg/mL) for 24 h. After a washing step with PBS, cells were detached using EDTA (0.02% in PBS), centrifuged for 5 min at 450× *g* (Centrifuge 5417 R, Eppendorf Austria GmbH, Vienna, Austria), and resuspended in PBS. Cell surface markers (i.e., brilliant violet 650 anti-human CD80 antibody, PE–cyanine5 anti-human CD86 antibody, APC–cyanine7 anti-human HLA-DR antibody) suspended in PBS were added to the cells, followed by an incubation period of 15 min. The cells were fixated using 2% formaldehyde and analyzed with a Cytek Aurora system (Cytek Biosciences, Fremont, CA, USA) equipped with the Cytek SpectroFlow^®^ 3.3.0 software. Untreated cells and cells treated with 1 µg/mL LPS served as cell control.

### 4.5. Statistical Analysis

Results presented are expressed as mean values ± standard deviation (SD). The statistical analysis was conducted using a one-way analysis of variance, followed by pairwise comparisons using the Holm–Šidák method to identify significant differences between groups. Differences were considered significant at levels of *p* < 0.05, *p* < 0.01, and *p* < 0.001.

## 5. Conclusions

M0 and iDCs were successfully differentiated from human monocytes, exhibiting cell-specific morphological and functional characteristics. We found that electroneutral sulfo-DIBMA nanodiscs induced minimal immune activation in these M0 and iDCs, as evidenced by lower ROS and cytokine production, along with reduced particle uptake and cell activation. On the contrary, anionic glyco-DIBMA nanodiscs triggered a strong immune response. In particular, we observed that iDCs matured upon incubation with glyco-DIBMA nanodiscs, expressing co-stimulatory and antigen-presenting molecules, which could potentially activate CD4 T cells. This possible capacity to modulate T-cell differentiation has significant therapeutic implications, as it can be exploited to develop more targeted therapies that have the potential to proactively regulate pro-inflammatory responses.

## Figures and Tables

**Table 1:** Mean Z-average [nm] and the corresponding polydispersity index (PDI) values as well as zeta-potential [mV] results of glyco- and sulfo-DIBMA (diisobutylene–maleic acid) nanodiscs in phosphate buffer saline (PBS) and Roswell Park Memorial Institute (RPMI) 1640 medium.

	Glyco-DIBMA Nanodiscs			Sulfo-DIBMA Nanodiscs		
	Size [nm]	PDI	Zeta potential [mV]	Size [nm]	PDI	Zeta potential [mV]
PBS	14.9 ± 0.3	0.307 ± 0.026	−15.5 ± 2.9	13.2 ± 1.2	0.262 ± 0.018	−3.5 ± 1.6
RPMI (0 h)	13.0 ± 0.5	0.215 ± 0.011	−12.6 ± 3.3	17.7 ± 1.0	0.325 ± 0.009	−2.3 ± 0.7
RPMI (24 h)	13.1 ± 0.3	0.250 ± 0.007	−12.7 ± 0.8	11.4 ± 0.3	0.191 ± 0.038	−2.0 ± 0.2

## Data Availability

Data is contained within the article.
